# A comparison of two methods for estimating odds ratios: Results from the National Health Survey

**DOI:** 10.1186/1471-2288-8-78

**Published:** 2008-11-25

**Authors:** Enayatollah Bakhshi, Mohammad R Eshraghian, Kazem Mohammad, Behjat Seifi

**Affiliations:** 1 Department of Biostatistics, School of Public Health and Institute of Public Health Research, Tehran University/Medical Sciences, Iran; 2 Department of Physiology, Medicine School, Tehran University/Medical Sciences, Iran

## Abstract

**Background:**

The practice of dichotomizing a continuous outcome variable does not make use of within-category information. That means the loss of information. This study compared two approaches in the modelling of the association between sociodemographic and smoking with obesity in adult women in Iran.

**Methods:**

We conducted a comparative study between two methods via an illustrative example, using data from the "National Health Survey in Iran (NHSI)" database. It included 14176 women aged 20–69 years. At first, body mass index(BMI) was treated as a continuous variable, OR_s _and 95 per cent confidence intervals were calculated using the "without dichotomizing" method. Then subjects were classified into obese (BMI ≥ 30 kg/m^2^) and nonobese (BMI < 30 kg/m^2^) and logistic regression model was used to estimate OR_s _and 95 per cent confidence intervals.

**Results:**

The odds ratio estimates changed only slightly over the two methods. But the "without dichotomizing" method provided shorter confidence intervals on the odds ratio parameters than dichotomizing method. All relative confidence interval lengths were greater than 1.15.

**Conclusion:**

If responses are continuous then the "without dichotomizing" method is certainly more useful than the "dichotomizing" method and leads to more precise estimation of odds ratios.

## Background

Over the past 20 years, the logistic regression model has become more common. The parameter in logistic regression has the interpretation of log odds ratio, which is easy for people such as physicians to understand. This model uses a categorical (dichotomous or polytomous) outcome variable. In many areas of research, the outcome data are continuous. Many researchers have no hesitation in dichotomizing a continuous variable but this practice does not make use of within-category information. Several investigators have noted the disadvantages of dichotomizing [1-9].

Although Goldwasser and Fitzmaurice [10] stated that a 'direct comparison of the logistic and linear regression coefficients is not meaningful since they have different interpretations, Moser and Coombs [11] provided a closed form relationship that allows a direct comparison between the logistic and linear regression coefficients. They also provided a procedure that allows the researcher to analyze the original continuous outcome without dichotomizing.

The aims of this paper are: (1) to demonstrate that the coefficient estimates from the "without dichotomizing (WDICH)" method have smaller variances and shorter confidence intervals than the dichotomizing (DICH) method; and (2) to find more efficient parameter estimates than logistic regression model for the association of sociodemographic and smoking with obesity by using cross-sectional data from the 1999–2000 National Health Survey in Iran.

## Methods

### Overview of WDICH method

The WDICH method overcomes some of the disadvantages of logistic regression model [11]. The linear regression model can be stated as follows:

$$y_{i}=x_{i}^{'}\beta +e_{i}$$

Where *e*_*i *_is random error term with mean 0 and variance *σ*^2 ^> 0; *e*_*i *_and *e*_*j *_are uncorrelated so that the covariance (*e*_*i*_, *e*_*j*_) = 0 for all *i*, *j*; *i *≠ *j*. Moser and Coombs supposed that the random terms *e*_*i *_follow a logistic distribution and explanatory variables ***x***_*i *_follow a discrete uniform distribution. They provided an estimate of the same odds ratio parameter as the DICH method, but without loss of information [11]. The estimates obtained from WDICH are more efficient than those from the logistic model [11]. They also carried out an extensive simulation study to evaluate the robustness of this conclusion to changes in the distributions of *e*_*i *_and ***x***_*i *_[11]. The reliability of these simulation results is assessed in this paper.

### Data set examined

The NHSI is a survey designed to gain comprehensive knowledge and information about health care problems and difficulties throughout in Iran, 1999–2000. Data from the NHSI were considered in this investigation. In this study, 14176 women, 8957 urban and 5219 rural aged 20–69 years were investigated. We excluded pregnant women from the analyses. This study is approved by the Ethic Committee of the Tehran University of Medical Sciences.

### Model variables

#### a)Response variable

Height and weight were measured rather than self-reported. BMI (Body Mass Index) was calculated as weight in kilograms divided by the square of the height in meters (kg/m^2^), and subjects were classified into obese (BMI ≥ 30 kg/m^2^) and nonobese (BMI < 30 kg/m^2^).

#### b) Independent variables

**i. Place of residence**: Urban (1) or Rural (0);

ii. Age (yr);

**iii. Education**: The total number of years of education;

**iv. Smoking status**: Smoker (1) or Nonsmoker (0);

**v. Marital status**: Married (1) or Non-married (0);

**vi. Economic index**: Economic index was defined as square meter of living place divided by number of household. Participants were classified by their economy index status into four classes: 1) low (economic index ≤ Quartile 1), 2) lower-middle (Quartile 1 < economic index ≤ Quartile 2), 3) upper-middle (Quartile 2 < economic index ≤ Quartile 3) and 4) high (economic index > Quartile 3).

### Statistical analysis

At first, BMI was treated as a continuous variable and is expressed as a function of place of residence, age, education, smoking, marital status and economic index using the WDICH method, OR_s _and 95 per cent confidence intervals were calculated. Then subjects were classified into obese (BMI ≥ 30 kg/m^2^) and nonobese (BMI < 30 kg/m^2^) and logistic regression model was used to estimate OR_s _and 95 per cent confidence intervals.

Two methods were compared with respect to relative confidence interval length of parameter estimates.

Analyses results were obtained using STATA (Version 8.0) and R (Version 2.0.1).

## Results

Distribution of age, BMI, education, marital status, economic index and smoking are shown in table 1 in order to make the data presentation complete. The mean BMI of urban women was 26.02 kg/m^2^(95 percent CI: 25.92–26.12). The rural women had a mean BMI 24.14 kg/m^2 ^(95 percent CI: 24.02–24.26).

**Table 1:** Characteristics of the analytical sample by place of residence in 14176 Iranian women, 1999–2000

**Variables**	**Rural (n = 5219)**	**Urban (n = 8957)**
Age, years(mean, sd^a^)	36.91 (13.58)	37.3(13.6)
BMI^b^(mean, sd)	24.14(4.64)	26.02(5.06)
Years of education(mean, sd)	2.59(3.42)	5.65(4.83)
Married (number)	3973	7234
Non-married (number)	1246	1723
Low economic index(number)	1655	2379
Lower-middle economic index(number)	1036	2271
Upper-middle economic index(number)	1280	2212
High economic index(number)	1248	2095
Smoker (number)	97	176
Non smoker(number)	5122	8781

^a^standard deviation

^b^Body mass index (weight (kg)/height(m)^2^).

Results in Table 2 were obtained from fitting Models in DICH and WDICH methods. DICH and WDICH produced different confidence intervals, although the odds ratios were similar. The odds ratio estimate from the WDICH method had smaller variances and shorter confidence intervals than the DICH method. The mathematical proof and simulation results are found in Moser and Coombs [11].

**Table 2:** Adjusted odds ratios for obesity and confidence intervals using two methods for the National Health Survey

**Covariates**	**Odds ratio**	**95% CI**^a ^ **(Without dichotomizing)**	**95% CI** **(dichotomizing)**	**Relative length of CI** **(dichotomizing/without dichotomizing)**
Place of residence	2.04(2.13)^b^	1.916–2.194	1.915–2.369	1.63
Age	1.03(1.02)	1.026–1.032	1.017–1.026	1.50
Years of education	0.99(0.98)	0.979–0.996	0.968–0.993	1.47
Smoking	0.69(0.65)	0.553–0.856	0.468–0.916	1.48
Marital status	1.24(1.48)	1.150–1.323	1.312–1.668	2.06
Lower-middle economy index	1.36(1.37)	1.246–1.475	1.206–1.554	1.52
Upper-middle economy index	1.31(1.29)	1.205–1.426	1.136–1.468	1.50
High Economy Index	1.29(1.25)	1.155–1.443	1.094–1.425	1.15

^a^confidence interval

^b^without dichotomizing(dichotomizing)

### Explanation of results from Table 2 (WDICH)

❑ Urban women had significantly higher odds of obesity than their rural counterparts (OR = 2.041, 95% CI: 1.916 – 2.914).

❑ Age was directly associated with obesity (OR = 1.03, 95% CI: 1.026–1.032).

❑ Education was inversely associated with obesity (OR = 0.99, 95% CI: 0.979–0.996).

❑ Non smoker women were more obese than smokers. Obesity odds ratio was 0.69 (95 percent CI: 0.553–0.856) for smoker women compared to non smokers.

❑ Married women had significantly higher odds of obesity than their non-married counterparts (OR = 1.24, 95% CI: 1.150 – 1.323).

❑ An association observed between economic index and obesity. Using low as the reference group, obesity odds ratios were 1.36 (95 percent CI: 1.246–1.475), 1.31 (95 percent CI: 1.205–1.426) and 1.29 (95 percent CI: 1.155–1.443) for the lower-middle, upper-middle and high groups respectively.

## Discussion

Dichotomizing the primary outcome variable may result in loss of information. We conducted a comparative study between two methods via an illustrative example, using data from the NHSI database. It included 14176 women aged 20–69 years. OR estimates and 95 per cent confidence intervals were calculated using both the DICH method and WDICH method. Overall, we obtained similar parameter estimates from DICH and WDICH methods. But the odds ratio estimate from the WDICH method had smaller variances and shorter confidence intervals than the DICH method. Our results indicated the improvement of the WDICH method over the DICH method because for all covariates the relative confidence interval length was greater than 1.15. Our results were consistent with the findings by Moser and Coombs [11] showing the greater efficiency of parameter estimates from WDICH method in comparison to DICH method.

In our study, there was a positive association between age and obesity. Our results are consistent with most studies [12-15].

In most studies, women with lower education were more obese than those with higher education. Our results were consistent with these studies [16-20].

We observed an inverse association between smoking and obesity. Most studies report that smoking is associated with lower relative weight [21-25]. Our findings are basically in line with these studies.

We found that non-married women were less likely to be obese than their married counterparts. Our results are consistent with most studies [26,27].

We found a statistically significant association between economic index level and obesity for women. Women with low level were leaner than those with other levels. Our findings are consistent with some study in developing countries [28].

One of the limitations of this study is the cross-sectional nature of the NHSI dataset. This means that we cannot draw definitive conclusions concerning the direction of causality. It is another limitation that physical activity and income were not used in our investigation. The other limitation in this study is that marital status could be categorized into legally married and non-married only.

Our study had several strengths. It was performed in a nationally representative sample of the Iranian women. Height and weight were actually measured rather than self-reported. It is well known that self-reports underestimate the prevalence of obesity [29,30].

## Conclusion

WDICH method is useful to estimate odds ratios and provides more efficient parameter estimates than DICH method when responses are continuous. When outcome is a continuous variable, it should not be treated as a binary variable.

## Abbreviations

BMI: body mass index; OR: odds ratio; NHSI: National Health Survey in Iran; WDICH: without dichotomizing; DICH: dichotomizing.

## Competing interests

The authors declare that they have no competing interests.

## Authors' contributions

EB, KM and MRE originated the idea for this study, did the research proposal, data analysis and prepared the manuscript. BS helped and edited the final version as the medical consultant. All authors read and approved the final manuscript.

## Pre-publication history

The pre-publication history for this paper can be accessed here:


